# A Novel Conductive Poly(3,4-ethylenedioxythiophene)-BSA Film for the Construction of a Durable HRP Biosensor Modified with NanoAu Particles

**DOI:** 10.3390/s16030374

**Published:** 2016-03-15

**Authors:** Fangcheng Xu, Shuaibin Ren, Yesong Gu

**Affiliations:** 1 Department of Chemical and Biochemical Engineering, Xiamen University, No. 422, Siming South Road, Xiamen 361005, Fujian, China; fcxu@xmu.edu.cn (F.X.); rht0517@126.com (S.R.); 2 Department of Chemical and Materials Engineering, Tunghai University, No.1727, Sec.4, Taiwan Boulevard, Xitun District, Taichung 40704, Taiwan

**Keywords:** bovine serum albumin, poly(3,4-ethylenedioxythiophene), nano Au particle, HRP biosensor, electron transfer mediator

## Abstract

In this study, we have investigated the contribution of bovine serum albumin (BSA) to the durability of the electrochemically synthesized poly(3,4-ethylenedioxythiophene) (PEDOT) film on a platinum (Pt) electrode. The electrode was capable to effectively adsorb the nano Au particles (AuNPs) to form a uniform layout, which was then able to immobilize the horseradish peroxidase (HRP) to construct a functional HRP/AuNPs/PEDOT(BSA)/Pt biosensor. Cyclic voltammetry was employed to evaluate the performance of the biosensor through the measurement of hydrogen peroxide. Our results revealed a satisfied linear correlation between the cathodic current and the concentration of H_2_O_2_. Furthermore, the addition of oxidized form of nicotinamide adenine dinucleotide, or NAD^+^, as the electron transfer mediator in the detection solution could dramatically enhance the sensitivity of detection by about 35.5%. The main advantages of the current biosensor are its durability, sensitivity, reliability, and biocompatibility.

## 1. Introduction

Since poly(3,4-ethylenedioxythiophene), or PEDOT, is a fascinating conductive polymer that exhibits advantages to other intrinsic conducting polymers, including good thermal and environmental stabilities [1], it has drawn considerable attention in fabricating various types of electrochemical biosensors [2,3,4,5,6]. Platinum (Pt) electrodes coated with conductive polymers display less background interference from unexpected components, but exhibit extremely high sensitivity towards the target [7,8,9]. PEDOT can be easily synthesized by either chemical or electrochemical polymerization approaches, and its well-defined surface morphology is adapted to immobilize biomolecules, such as proteins and oligonucleotides, for the construction of biosensors. Upon a proper doping, the conductivity of PEDOT may be modulated to be suitable for various applications.

Recent publications have suggested that the PEDOT-modified biosensor has advantages for the detection of DNA and other biomarkers [8,10,11,12,13]; however, the durability of the PEDOT film is still a major challenge for practical measurements. It has been often noticed that the electrochemical synthesized PEDOT film on the Pt electrode suffers from fracturing during preparation and operation, which influences the long-term performance of the electrode. We ascribed the fracture to the formation of short PEDOT fragments in the absence of a template as well as the surface tension between the rather hydrophilic platinum and the hydrophobic PEDOT film. It has been reported that the addition of polystyrene sulfonates (PSS) may improve the adhesion property of PEDOT film on the electrode [14]; however, we found that gold nanoparticles (AuNPs) were unable to be successfully captured by the PEDOT:PSS film and therefore failed to immobilize biomolecules with AuNPs. On the other hand, we have previously proposed that bovine serum albumin (BSA) is able to serve as the template for the electrochemical synthesis of polyaniline (PANI) and stabilize the PANI film [15]. BSA was also employed as the template in the enzymatic synthesis of water-soluble PANI [16]. Furthermore, BSA is a lysine-rich protein that may provide extra amino groups for the immobilization of protein. In this study, we expected that BSA might provide similar functions for the polymerization of PEDOT that could improve the adhesion property of the PEDOT film on the electrode as well as offer functional groups to effectively immobilize proteins for the construction of enzyme-based biosensors.

AuNPs have been widely employed to immobilize proteins and DNA probes for the fabrication of biosensors with specific applications [17,18]; however, we have noticed that AuNPs cannot attach to the PEDOT(PSS) film with good stability, which can possibly be attributed to the electric repulsion between the exposed anionic PSS and the AuNPs surrounded by anionic ions. In this study, we have investigated the contribution of BSA to the electrochemical synthesis of a durable PEDOT film, where AuNPs were able to attach to the PEDOT(BSA) film to form a uniform distribution effortlessly. Horseradish peroxidase (HRP) was then immobilized to construct a functional HRP/AuNPs/PEDOT(BSA)/Pt biosensor without further chemical modification and using crosslinking agents. Cyclic voltammetry (CV) is one of the most widely employed techniques and is able to provide information about the redox potential and electrochemical reaction occurring on the electrode surface. In this study, CV was used to evaluate the performance of the biosensor through the measurement of hydrogen peroxide (H_2_O_2_). Furthermore, we have explored the influence of the oxidized form of nicotinamide adenine dinucleotide (NAD^+^) as the electron transfer mediator on the measurement and found that the sensitivity of detection was dramatically enhanced. This study has proposed a novel strategy to fabricate a biosensor with useful properties in terms of durability, sensitivity, reliability, and biocompatibility.

## 2. Experimental Section

### 2.1. Chemicals

3,4-ethylenedioxythiophene (EDOT), HRP, and BSA were purchased from Sigma-Aldrich Corp. (St. Louis, MO, USA). Lithium perchlorate trihydrate (LiClO_4_) and H_2_O_2_ (35%, *v*/*v*) were obtained from Merck KGaA (Darmstadt, Germany). Ultrapure water treated by a Milli-Q Plus system from EMD Millipore (Darmstadt, Germany) was used to prepare all the solutions. All other reagents used for buffers and solutions were purchased from various commercial sources and were of analytical grade.

### 2.2. Electrochemical Apparatus

A PC-controlled CHI621B electrochemical analyzer (CHI instruments, Austin, TX, USA) was employed to run cyclic voltammetric experiments for electrode fabrication and hydrogen peroxide measurement. All experiments were performed in a miniature electrochemical cell using a modified Pt electrode (working area: 0.1912 cm^2^) as the working electrode, a platinum wire as the auxiliary electrode, and an Ag/AgCl (3 M NaCl) electrode as the reference electrode. All experiments were carried out at room temperature and performed in 0.1 M PBS (pH 6.2) under a nitrogen atmosphere.

### 2.3. Electrochemical Synthesis of PEDOT(BSA) Composite for AuNPs/PEDOT(BSA) Electrode

To electrochemically synthesize the PEDOT(BSA) composite film on the Pt working electrode, a Pt wire with a diameter of 0.6 mm was submerged into a solution containing 0.1 M LiClO_4_, 0.01 M EDOT, and 1.0 mg/mL BSA, while the potential was swept from 0.0 to 1.0 V for 10 cycles under an ambient condition. BSA was expected to sever as the template for PEDOT synthesis and to strengthen the stability of PEDOT film. The amount of BSA was quantified according to Bradford assay with a Genesys 2 UV-vis spectrophotometer (Rochester, NY, USA).

AuNPs with a mean diameter of 16 nm was prepared by reducing HAuCl_4_·4H_2_O with sodium citrate in a aqueous solution at 100 °C for 1.5 h according to the literature [19]. After drying and nitrogen treatment, the fashioned PEDOT(BSA)/Pt electrode was then immersed in the above AuNPs solution. In order to deposit AuNPs onto the PEDOT film, a +0.4 V potential was applied on the electrode for 10 min, and the anionic AuNPs stably attached to the positively charged PEDOT film. The surface morphologies of the electrodes were visualized by an ABT-150S SEM (TOPCON Corp., Tokyo, Japan).

### 2.4. Enzyme Immobilization

The constructed AuNPs/PEDOT(BSA)/Pt electrode was incubated in a 5 mg/mL HRP solution for 30 min under RT °C to allow HRP molecules to be immobilized in order to fabricate the HRP/AuNPs/PEDOT(BSA)/Pt electrode. All resulting electrodes in this study were washed with 0.1 M PBS (pH 6.2) at least three times and stored at 4 °C for further characterizations. HRP activity was assessed by 1-Step^™^ ABTS method (PIERCE Chemical Co., Rockford, IL, USA) according to the manufacturer’s procedures.

### 2.5. Electrochemical Measurements

The catalytic mechanism of immobilized HRP to the reduction of H_2_O_2_ has already been proposed [20]. The reductions of H_2_O_2_ on electrodes were quantified with cyclic voltammetry in 0.1 M PBS buffer (pH 6.2). The buffer had undergone deoxygenation with highly pure nitrogen for 30 min before a certain amount of H_2_O_2_ was added. During the calibration, pure nitrogen gas was gently purged on the surface of the sample solution to create an anaerobic atmosphere. The CVs were recorded from −0.2 to 0.8 V at the scan rate of 50 mV. All experiments were carried out at room temperature (25 °C).

## 3. Results and Discussion

### 3.1. Enhancing the Stability of PEDOT Film with BSA

**Figure 1:**
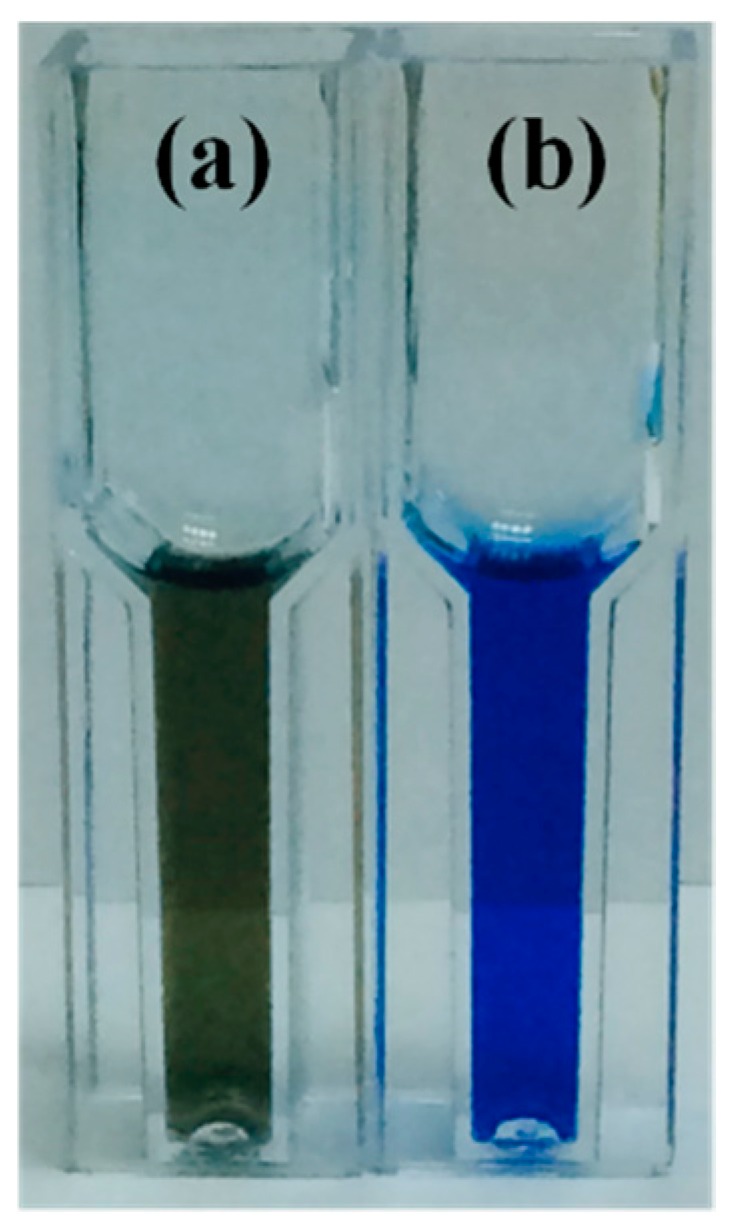
The Bradford protein assay after submerging the Pt strips in a 50 mM PBS solution (**a**) in the absence and (**b**) in the presence of 1.0 mg/mL of BSA.

In order to improve the stability of PEDOT film on the Pt electrode, we investigated the contribution of BSA by adding it to the reaction solution during the electrochemical polymerization of PEDOT. For routine bioassay procedures, BSA is an inert protein that is commonly used as a blocking agent to occupy the solid surface through its non-specific binding ability. In this study, we have proved the adsorption of BSA to platinum by immersing a Pt strip in a BSA solution followed by submerging the strip in a Bradford protein assay solution (Figure 1).

**Figure 2:**
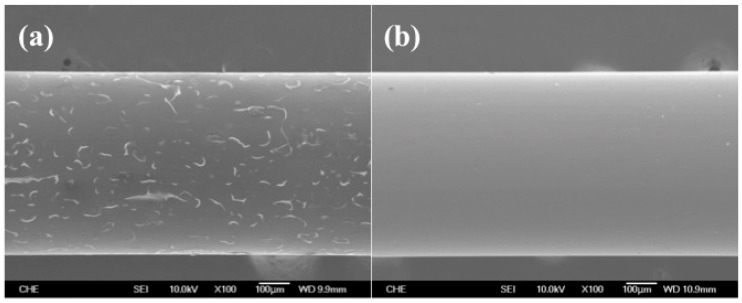
SEM images of (**a**) PEDOT/Pt electrode after one measurement and (**b**) PEDOT(BSA)/Pt electrode after 10 measurements.

The poor adhesion of the PEDOT film on the Pt electrode was presumably due to the fact that the surface of Pt was relatively hydrophilic, but the EDOT oligomers and PEDOT were considerably hydrophobic in an aqueous solution; we therefore expected that the attachment of BSA might neutralize the surface properties of Pt electrode and be beneficial to the durability of the PEDOT(BSA) film. BSA might also serve as the template for the polymerization of PEDOT and modify the microstructure of the PEDOT film, which was similar to our previous discoveries for the electrochemical and enzymatic synthesis of conductive polyaniline in the presence of BSA [7,16]. As expected, the SEM images shown in Figure 2 indicated that the PEDOT film cracked badly after one time measurement, but no cracks were detectable in the PEDOT(BSA) film after multiple measurements, suggesting that the adhesion and durability of PEDOT(BSA) film on the Pt electrode were dramatically improved in comparison with those of the PEDOT film.

### 3.2. The Construction of a AuNPs/PEDOT(BSA)/Pt Electrode

**Figure 3:**
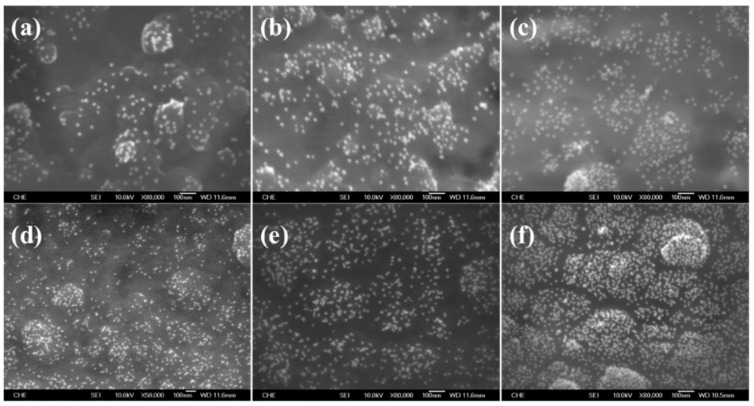
The SEM images of the surface morphologies of AuNPs/PEDOT(BSA)/Pt electrodes. (**a**–**c**): the electrodes were pretreated with −0.4 V, no treatment, and +0.4 V, respectively, in a PBS solution (pH = 6.2) for 10 min before being soaked in the AuNPs solution for 60 min; (**d**–**f**): the electrodes were pretreated with +1 V for 10 minutes before being soaked in the AuNPs solution for 10, 30, and 60 min, respectively.

In order to fabricate a HRP/AuNPs/PEDOT(BSA)/Pt biosensor, we first investigated the immobilization of AuNPs on the PEDOT(BSA) film. Theoretical calculation revealed that the Au-S bonds might hold the major portion of electrostatic but partially covalent interaction [21]. Accordingly, we believed that the electrostatic attraction played a very significant role in the deposition of AuNPs on the PEDOT(BSA) film. Figure 3 shows SEM images of PEDOT(BSA) after AuNPs deposition, where the PEDOT(BSA)/Pt electrode went through a potentiostatic procedure before being soaked in a PBS buffer solution containing AuNPs. The results clearly indicated that the positively charged PEDOT(BSA) film (+0.4 V) could immobilize many more AuNPs than that of a negatively charged PEDOT(BSA) film (−0.4 V), and the higher the positive potential applied the more AuNPs were immobilized. This was because our homemade AuNPs were surrounded by anions in the sodium citrate aqueous solution based on the negative value of the zeta potential. In addition, the longer the soaking time the more AuNPs were captured on the PEDOT(BSA) film, as shown in Figure 3d–f.

### 3.3. The Immobilization of HRP and Its Bioactivity Measurement

**Figure 4:**
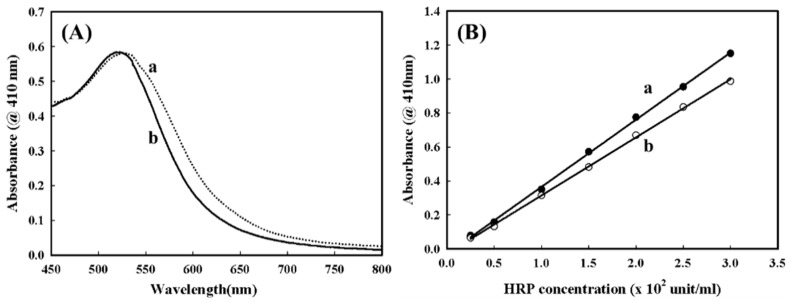
(**A**) The UV-vis spectrum of a PBS solution containing (**a**) AuNPs and (**b**) AuNPs-HRP; (**B**) the standard curve for the activity of (**a**) HRP and (**b**) AuNPs-HRP.

HRP, a heme-containing peroxidase, has been the most comprehensively studied for fabricating enzyme-based biosensors since it catalyzes the oxidations of a wide variety of organic and inorganic substrates. To construct an enzyme-based biosensor, HRP is commonly immobilized on the solid surface of an electrode through crosslinking agents, such as N-hydroxysuccinimide (NHS) plus carbodiimide (EDC) or glutaraldehyde. However, the immobilization efficiencies of these procedures are often time-consuming, with less reliability in practice. Amino acid sequence analysis has revealed that HRP contains several cysteine residues that are available for the interactions with AuNPs to facilitate the stable Au–S bonds, even though they may have already been in the form of disulfide bonds [22]. The immobilization of HRP slightly increased the diameter of the AuNPs by several nanometers and did not significantly alter the homogeneity of AuNPs (Figure 4A). In addition, Figure 4B demonstrates that the HRP immobilized with AuNPs possessed good bioactivity with a slight reduction in comparison to that of pure native HRP. Recently, we have noticed that the AuNPs hardly attracted the bare Pt electrode, but AuNPs could be effectively captured by the PEDOT(BSA) film with uniform distribution and good stability; therefore the construction of an HRP/AuNPs/PEDOT(BSA)/Pt biosensor was feasible.

As has been mentioned above, the HRP was easily captured by AuNPs and retained most of its bioactivity. After successfully constructing the AuNPs/PEDOT(BSA)/Pt electrode, the electrode was soaked in a PBS buffer solution (pH 6.2) containing 5 mg/mL HRP for 30 min, at room temperature with gentle agitating. After washing the electrode with PBS buffer three times, the HRP activity remaining in the flash solution was almost undetectable. On the other hand, the electrode displayed good HRP activity, approximately 0.1569 unit/cm^2^ according to the standard curve of HRP activity or 1.11 × 10^13^ active HRP molecules/cm^2^ for the effective working area of 0.1912 cm^2^. Indeed, the HRP/Au/PEDOT(BSA)/Pt electrode was biochemically functional.

### 3.4. Electrochemical Responses to H_2_O_2_ with Modified Electrodes

**Figure 5:**
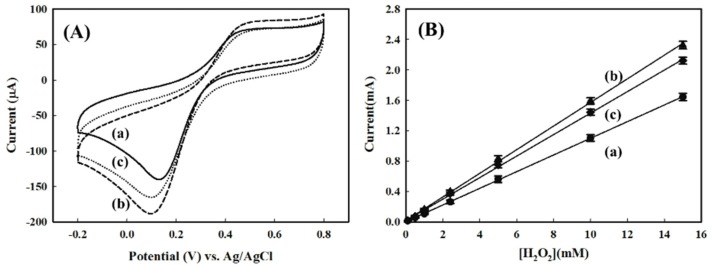
(**A**) CV profiles of electrodes in response to 1.0 mM of H_2_O_2_: (**a**) PEDOT(BSA)/Pt, (**b**) AuNPs/PEDOT(BSA)/Pt, and (**c**) HRP/AuNPs/PEDOT(BSA)/Pt, respectively; (**B**) The linear correlations of peak current and concentration of H_2_O_2_ for the three corresponding electrodes, (**a**) PEDOT(BSA)/Pt; (**b**) AuNPs/PEDOT(BSA)/Pt and (**c**) HRP/AuNPs/PEDOT(BSA)/Pt, respectively. Each line was obtained from one single electrode with three experiments for every concentration of H_2_O_2_.

Figure 5A shows the CV of electrodes at different fabricating stages in response to 1.0 mM of H_2_O_2_. It was interesting to note that both PEDOT(BSA)/Pt and AuNPs/PEDOT(BSA)/Pt electrodes exhibited distinguishable reduction peaks of H_2_O_2_ at the potential of 0.13 V and 0.10 V, respectively, with a scan rate of 50 mV/s. These results indicated that both PEDOT and AuNPs possessed the electrocatalytic activities for the reduction of H_2_O_2_, although PEDOT and AuNPs did not show any ABTS activity. In addition, the attachment of AuNPs might not only increase the reactive surface area but also enhance the electron transfer. On the other hand, the CV profile for the HRP/AuNPs/PEDOT(BSA)/Pt electrode displayed a similar reduction peak to that of the AuNPs/PEDOT(BSA)/Pt electrode, but with a reduced peak current. The cathodic currents for AuNPs/PEDOT(BSA)/Pt and HRP/AuNPs/PEDOT(BSA)/Pt are 145.1 μA and 132.6 μA, respectively, in response to 1 mM of H_2_O_2_. The three electrodes all exhibited linear correlations of the cathodic current to the concentration of H_2_O_2_, as shown in Figure 5B, and the criteria for their performances are listed in Table 1.

### 3.5. Enhancing the Electrochemical Performance of HRP/AuNPs/PEDOT(BSA)/Pt with NAD^+^

Nevertheless, we expected that HRP/AuNPs/PEDOT(BSA)/Pt could have an advantage over other electrodes due to the biochemical specificity of HRP. According to the HRP-catalyzed mechanism for the reduction of H_2_O_2_, the oxidized intermediate form of HRP required electrons from the electrode.

**Figure 6:**
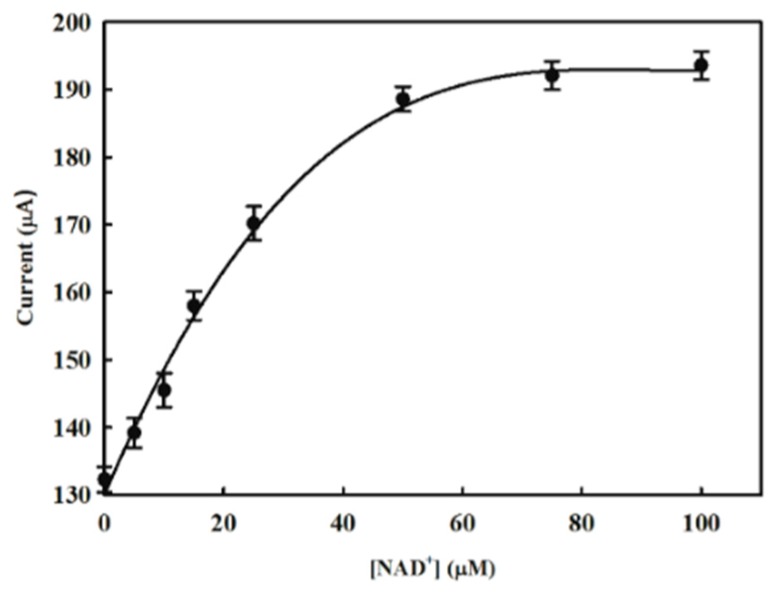
The effect of various NAD^+^ concentration in PBS buffer (pH 6.2) with 1.0 mM H_2_O_2_ on the performance of HRP/AuNPs/PEDOT(BSA)/Pt with a scan rate of 50 mV·s^−1^. The curve was obtained from one single electrode with three experiments for every concentration of NAD+.

**Figure 7:**
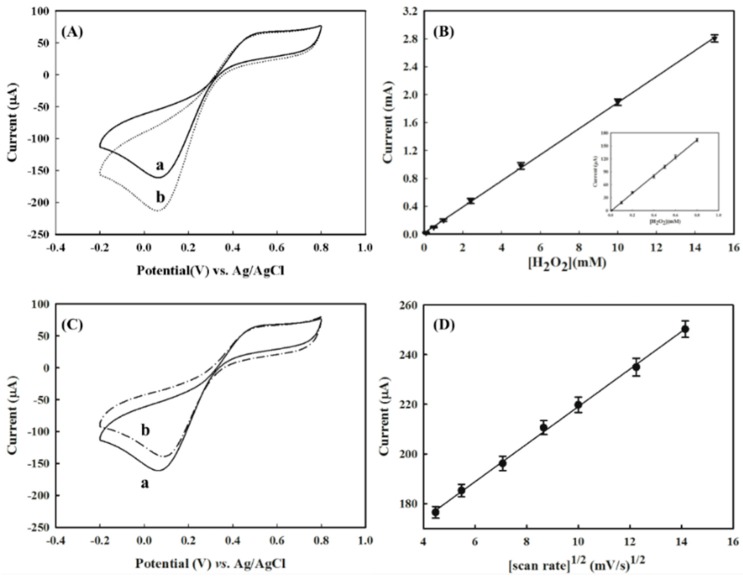
(**A**) The CV profiles of HRP/AuNPs/PEDOT(BSA)/Pt electrodes in response to 1.0 mM of H_2_O_2_ in 0.1 M PBS buffer (pH 6.2) (**a**) without and (**b**) with the addition of 50 μM NAD^+^; (**B**) the linear correlation of the cathodic current in response to the concentration of H_2_O_2_ in the presence of 50 μM of NAD^+^, where the inset displays the linear correlation for the low concentration of H_2_O_2_; (**C**) the CV profiles of HRP/AuNPs/PEDOT(BSA)/Pt electrodes in response to 1.0 mM of H_2_O_2_ (**a**) without and (**b**) with the addition of 50 μM NADH; (**D**) the correlation of cathodic current in response to 1.0 mM of H_2_O_2_ in the presence of 50 μM NAD^+^ with a different scan rate.

In order to overcome the ineffective ET from the electrode to the active center of the HRP intermediates, we investigated the contribution of the ET mediator, NAD^+^, to the performance of the HRP/AuNPs/PEDOT(BSA)/Pt electrode, since the NADH formed from the reduction of NAD^+^ on the electrode may be further oxidized by the HRP intermediate and could assist the ET based on the proposed mechanism [23]. We found that an increase in the NAD^+^ concentration could significantly increase the cathodic current, and the increment reached a plateau at about 50 μM of NAD^+^ (Figure 6). Figure 7A demonstrated that the addition of 50 μM NAD^+^ resulted in a dramatic increase in the cathodic current of about 42.3% in response to 1 mM of H_2_O_2_, and a good correlation between peak current and the potential applied was also obtained in the presence of NAD^+^ (Figure 7B). By comparison, the NAD^+^ caused only 11.2% and 15.3% improvement for the performance of AuNPs/PEDOT(BSA)/Pt and PEDOT(BSA)/Pt electrodes, respectively. The influence of NAD^+^ on the performance of different electrodes is summarized in Table 1. The sensitivity of 974.8 μA·mM^−1^·cm^−2^ for sensing H_2_O_2_ with the current electrode is higher than other published electrodes, such as 249 [24], 0.5638 [25], 8.44 [26], and 8.552 [27] μA·mM^−1^·cm^−2^. On the other hand, the addition of the reduced form of NAD, NADH, did not offer any beneficial effect on the cathodic current, as shown in Figure 7C.

**Figure 8:**
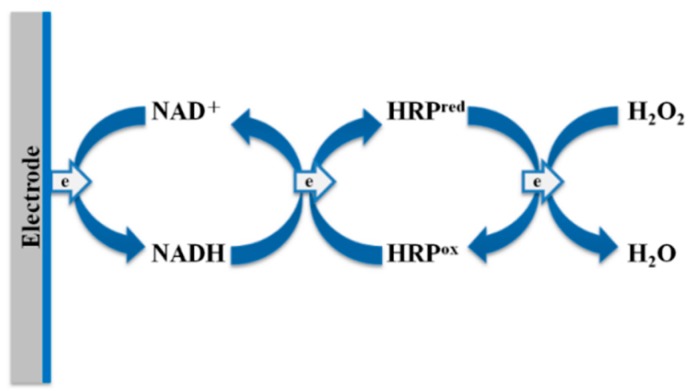
The course of electron transfer in the electrode with the aid of NAD^+^.

Line c in Figure 5A is the typical CV profile for the electron transfer reaction followed by a chemical reaction, which was characterized by variations from the reversible behavior and equivalent redox peak currents. In this study, the reaction was represented by the reduction of hydrogen peroxide that was catalyzed by HRP. For this type of electrochemical mechanism, the irreversible behavior was dependent not only on the rate of chemical reaction that relied on the catalytic activity of HRP, but also on the time scale of the experiment, which was reciprocal to the scan rate of CV. The addition of NAD^+^ accelerated the ET from the electrode to the active site of HRP and speeded up the regeneration of the active form of HRP. As shown in Figure 6, while the concentration of NAD^+^ exceeded approximately 50 μM, the determining effect of the chemical reaction on the CV profile became insignificant. On the other hand, Figure 7D demonstrated that further increasing the scan rate or reducing the time scale of the experiment caused the cathodic current to rise proportionally, which might imply that the cathodic current was diffusion controlled. Accordingly, the effective ET cascade occurring at the electrode is sketched in Figure 8.

## 4. Conclusions

**Figure 9:**
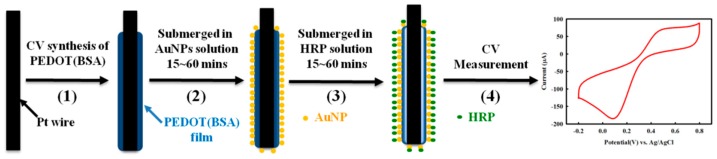
The procedure for the fabrication of HRP biosensor: (**1**) electrochemical synthesis of a PEDOT(BSA) film; (**2**) capturing the AuNPs by submerging the electrode in an AuNPs solution for 15~60 min; (**3**) immobilization of HRP by submerging the electrode in a HRP solution; (**4**) CV measurement of H_2_O_2_.

AuNPs have been used to immobilize biomolecules containing thiol function groups, such as DNAs, peptides, and proteins, through the stable Au–S formation. However, for common materials applied for electrodes such as Pt, we have noticed that AuNPs have difficulty attaching to Pt [23]. Interestingly, AuNPs were found to be easily captured by a PEDOT-modified electrode, but the PEDOT film is very unstable during the following modification and measurement procedure (Figure 2). Recently, we developed a PEDOT dual film to fabricate a durable DNA biosensor, where a durable PEDOT(PSS) layer was synthesized first and was then covered by a PEDOT layer, both of which were carried out by the electrochemical approach [28]. As a result, AuNPs were able to firmly attach to the PEDOT film effortlessly and could entrap the DNA probe modified with a thiol group. In this study, we have proposed an even simpler procedure for modifying the Pt electrode with a durable PEDOT(BSA) film and then AuNPs to form a fairly uniform distribution in order to fabricate an enzyme-based biosensor (Figure 9). Here, BSA functions as a surface buffer between the slightly hydrophobic PEDOT film and the slightly hydrophilic Pt electrode, and served as the template for PEDOT synthesis as well. Likewise, the AuNPs were able to immobilize HRP simply by submerging the electrode into a HRP solution to construct the HRP/AuNPs/PEDOT(BSA)/Pt electrode. The electrode was extremely durable and was able to sustain more than 20 repeat measurements without a significant decline in the signal. In addition, the oxidized form of ET mediator NAD^+^, but not the reduced form NADH, made a major improvement to the performance of HRP/AuNPs/PEDOT(BSA)/Pt electrode. In conclusion, we have presented a simple, durable, and sensitive enzyme-based biosensor modified with PEDOT(BSA) and AuNPs.

## Figures and Tables

**Table 1:** The responses of electrodes to 1.0 mM of H_2_O_2_ with cyclic voltammetry in the presence or absence of NAD^+^.

**Electrode ^a^**	**Peak Current (μA) ^b^**		**Sensitivity (μA·mM^−1^·cm^−2^) ^b^**	
	**No NAD^+^**	**50 μM NAD^+^**	**No NAD^+^**	**50 μM NAD^+^**
PEDOT(BSA)/Pt	114.3	127.1 (11.2%)	572.3	628.8 (9.9%)
AuNPs/PEDOT(BSA)/Pt	145.1	167.3 (15.3%)	786.2	871.2 (10.8%)
HRP/AuNPs/PEDOT(BSA)/Pt	132.6	188.7 (42.3%)	719.4	974.8 (35.5%)

**^a^**: The working area for all electrodes was approximately 0.1912 cm^2^; **^b^**: The value in parentheses is the percentage increment of the peak current and sensitivity of the same electrode in response to 1.0 M of H_2_O_2_ in the presence of 50 μM NAD^+^ in comparison with that in the absence of NAD^+^, for regeneration to its native active form. The unexpected decline for the signal of HRP/AuNPs/PEDOT(BSA)/Pt electrode possibly resulted from the immobilization of HRP on the AuNPs, which led to the slower electron transfer (ET) from the electrode to the active center of HRP that was critical for further reduction of H_2_O_2_.
